# Antitumor Efficacy of Human Monocyte-Derived Dendritic Cells: Comparing Effects of two Monocyte Isolation Methods

**DOI:** 10.1186/s12575-018-0069-6

**Published:** 2018-02-02

**Authors:** Graça S Marques, Zélia Silva, Paula A. Videira

**Affiliations:** 1 CEDOC, NOVA Medical School/Faculdade de Ciências Médicas, Universidade NOVA de Lisboa, Lisbon, Portugal; 2 UCIBIO, Departamento Ciências da Vida, Faculdade de Ciências e Tecnologia, Universidade NOVA de Lisboa, Lisbon, Portugal; 3 CDG & Allies – Professionals and Patient Associations International Network (CDG & Allies – PPAIN), Faculdade de Ciências e Tecnologia, Universidade NOVA de Lisboa, Lisbon, Portugal

**Keywords:** Monocyte-derived dendritic cells (MoDCs), Immunomagnetic cell isolation, T-cell cytotoxicity, Cancer vaccines

## Abstract

**Background:**

Dendritic cells (DCs), which can be used as anti-cancer vaccines, are generally obtained in vitro from isolated CD14^+^ monocytes (MoDCs). This generates high cell numbers and allows instructing DCs to guarantee effective antitumor responses. However, the impact of the monocyte isolation step in the antitumor effectiveness of the generated MoDCs is still unknown. Here, we compared the most used immunomagnetic technologies for monocyte isolation: magnetic activated cell sorting (MACS) from Miltenyi Biotec and EasySep from STEM CELL.

**Results:**

MACS technology allowed a higher monocyte yield and purity and, by flow cytometry, monocytes displayed higher size and lower granularity. In the resting state, EasySep_MoDCs showed a higher basal expression of HLA-DR, and no significant response to stimulation by LPS and TNF-α. When stimulated with whole tumor cells lysates, both MoDCs expressed similar levels of maturation and co-stimulatory markers. However, when cultured with autologous T cells, MACS_MoDCs induced significantly higher IFN-γ secretion than EasySep_MoDCs, indicating a stronger induction of Th1 cell response profile. Concordantly, T cells induced by MACS_MoDCs also showed a higher release of cytotoxic granules when in contact with tumor cells.

**Conclusions:**

Overall, both the MACS and the EasySep isolation immunomagnetic technologies provide monocytes that differentiate into viable and functional MoDCs. In our experimental settings, resting EasySep_MoDCs showed a higher basal level of maturation but show less responsivity to stimuli. On the other hand, MACS_MoDCs, when stimulated with tumor antigens, showed better ability to stimulate Th1 responses and to induce T cell cytotoxicity against tumor cells. Thus, monocyte isolation techniques crucially affect MoDCs’ function and, therefore, should be carefully selected to obtain the desired functionality.

## Background

Dendritic cells (DCs) are responsible for initiating several immune responses [1]. They have three main roles: to capture antigens, to migrate to T cell areas of lymphoid organs where they present antigens to T cells, and to induce expansion of antigen-specific T cell clones and their effector functions [2]. In their resting state, DCs are immature and they capture and present antigens [3]. The antigen uptake together with stimuli such as pro-inflammatory cytokines induces maturation, and DCs initiate a complex set of mechanisms, that leads to the upregulation of the major histocompatibility complex (MHC) class I and II and of co-stimulatory molecules, to activate T cells. Immature or not fully mature DCs may induce immune tolerance, leading to the downregulation of the response. Therefore, in the context of antitumor DC vaccination, a full maturation of DCs is highly required to induce effective antitumor immune responses, that is, subsequent proliferation of activated T cells that can kill tumor cells in an antigen-dependent manner [4–7].

Inopportunely, isolating human DCs for research or clinical purposes is difficult, due to the lack of a specific marker, and its low concentration in peripheral blood (~ 0.01% of all blood cells). To overcome this problem, high amounts of human DCs are generated in vitro from precursors, CD14^pos^ (CD14^+^) monocytes or CD34^+^ cells, isolated from peripheral blood [7]. Monocyte-derived DCs (MoDCs) are by far the most used in recent DC vaccine formulations [8] (ClinicalTrials.gov identifiers: NCT02533895, NCT02159937). The methods for monocyte isolation are either immunomagnetic separation or plastic adherence. The latter relies on the capacity of monocytes to adhere to plastic, but it is not specific and is only appropriate for assays where cell purity is not a major concern [9, 10]. To obtain high cell purity, the immunomagnetic separation is preferred and two of the widely used immunomagnetic separation technologies are magnetic activated cell sorting (MACS) technology (Miltenyi Biotec) and EasySep technology (StemCell Technologies). Both frequently use positive selection where leukocytes are incubated with magnetic beads linked to an antibody that recognizes the CD14 antigen. After incubation with the anti-CD14 antibodies combined with magnetic beads, the leukocytes are subjected to a magnetic field that retains the beads together with the CD14^+^ monocytes that are linked to them. The main differences between technologies concern the reagents that are used. Namely, MACS technology is based on the use of MACS microbeads, i.e. superparamagnetic particles coupled to anti-CD14 monoclonal antibodies, and columns. EasySep technology is column-free and uses tetrameric antibody complexes recognizing CD14 and dextran-coated magnetic particles.

In this study, we aimed to compare MACS and EasySep technologies regarding their efficiency to isolate functional monocytes and the downstream impact on the antitumor functionality of the derived MoDCs. Therefore, for each donor, we used both technologies in parallel to isolate monocytes and differentiated them into MoDCs, using the gold standard protocol with granulocyte/macrophage colony-stimulating factor (GM-CSF) and interleukin (IL)-4. The obtained MoDCs were assessed for the expression of antigen presentation and co-stimulatory molecules and for their efficacy to induce T cell-mediated anti-tumor responses. MoDCs were loaded with whole tumor cell lysates and used to stimulate autologous T cells, which were then cultured with tumor cells to assess their cytotoxic activity. Our data show that DCs derived from monocytes isolated with MACS or EasySep technologies, hereafter named MACS_MoDCs or EasySep_MoDCs, are both functional and can be activated by tumor antigens. However, MACS_MoDCs show lower basal maturation state, and when stimulated lead to a significantly higher production of IFN-γ by T cells, consistent with a higher capacity to instruct T cell cytotoxicity against tumor cells.

## Methods

### Cells, Media and Reagents

Leukocytes were cultured in RPMI-1640 media supplemented with 10% fetal bovine serum (FBS), 2 mM Glutamax, 100 μg/mL penicillin/streptomycin, 1% non-essential amino acids and 1% sodium pyruvate, purchased from Gibco (Paisley, Scotland, UK). The HLA-A*02:01 positive breast cancer cell line MCF-7 was obtained from American Type Culture Collection and was cultured in DMEM media supplemented with 10% FBS, 2 mM Glutamax and 100 μg/mL penicillin/streptomycin. All cultures were performed at 37 °C, in a humidified atmosphere of 5% CO_2_. Cell freezing media consisted in RPMI with 20% FBS and 10% dimethyl sulfoxide (DMSO; Sigma Chemical Co, St. Louis, MO, USA). IL-4 was purchased from R&D Systems (Minneapolis, MN, US), GM-CSF, IL-2, IL-7 and tumor necrosis factor (TNF)-α was from Miltenyi Biotec (Bergisch Gladbach, Germany), and *Escherichia coli* lipopolysaccharide (LPS) was from Sigma-Aldrich (St. Louis, Mo, USA).

### Cell Counting and Viability Examination

Cells were counted using a Neubauer chamber, following staining with trypan blue. Cell viability was also evaluated by flow cytometry, after staining with 7-Aminoactinomycin D (7AAD) (BD Biosciences, NJ, USA).

### Isolation of Peripheral Blood Mononuclear Cells

Peripheral blood mononuclear cells (PBMCs) were obtained from leuko-platelet concentrates from healthy donors, from the Portuguese Blood and Transplantation Institute (Instituto Português do Sangue e da Transplantação - IPST); and approval from the institutional ethical committee was previously obtained. PBMCs were isolated by density gradient centrifugation using Biocoll (Biochrom, Cambridge, United Kingdom), and then further washed to improve platelet removal. Each PBMCs sample was divided and processed in parallel with both immunomagnetic separation kits, as described below. HLA typing was performed and only donors with an HLA-A*02:01 profile were selected for the cytotoxicity assays.

### Isolation of CD14^+^ Monocytes Using CD14 MicroBeads from Miltenyi – MACS Technology

Monocyte isolation using the positive immunomagnetic selection kit from Miltenyi Biotec was performed according to the manufacturer’s instructions and as described [11, 12]. PBMCs were resuspended in phosphate-buffered saline (PBS) buffer, pH 7.2, containing 0.5% bovine serum albumin (BSA), and 2 mM ethylenediamine tetraacetic acid (EDTA); and incubated with CD14 microbeads (20 μL per 10^7^ cells) during 15 min at 4 °C. The cell suspension was loaded onto an LS magnetic column (Miltenyi Biotec) placed in the magnetic field of a MACS Separator (MIDIMACS) and rinsed three times with buffer. At this point, the CD14-positively labeled cells were retained in the magnetic field, while the negative cells were eluted. The column was then removed from the magnetic field, followed by the elution of the CD14^+^ fraction. Cell fractions were washed: CD14 cells were cultured and CD14^neg^ (CD14) cells were frozen.

### Isolation of CD14^+^ Monocytes Using EasySep Human CD14 Selection Kit from StemCell – EasySep Technology

Monocyte isolation using the positive selection kit from StemCell Technologies (Vancouver, BC, Canada) was performed according to the manufacturer’s instructions. Briefly, PBMCs were resuspended in PBS with 2% FBS and 1 mM EDTA and magnetically labeled in a two-step process. Firstly, PBMCs were incubated for 15 min at room temperature with Positive Selection Cocktail, tetrameric antibodies complexes (TAC) that recognize both CD14, and dextran. Then, dextran-coated EasySep Magnetic Nanoparticles were added and incubated 10 min at room temperature to allow them to bind to the TAC particles. The tube with the mixture was placed into an EasySep Magnet and incubated for 5 min, after which it was inverted to pour off the supernatant. At this point, magnetically labeled CD14^+^ cells remain inside the tube and were resuspended in buffer. The supernatant was re-incubated twice with the magnet and the remaining CD14^+^ cells were harvested and cultured and the CD14^−^ cells were frozen.

### Generation and Maturation of MoDCs

Monocytes isolated by either one of the previously described methods were resuspended at a density of 1 × 10^6^ cells/mL in culture media supplemented with 750 U/mL IL-4 and 1000 U/mL GM-CSF. The cell culture was plated in 6-well tissue culture plates and incubated for 7 days. Every 2 days, half of the culture media was replaced by fresh media supplemented with cytokines. For the maturation of MoDCs, culture media was supplemented with TNF-α (1000 IU/mL) and LPS (50 μg/mL).

### Loading MoDCs with Tumor Antigens

Lysates of cancer cells were obtained by four sequential freeze-thaw cycles (− 80 ° and 37 °C). The cell debris was removed by centrifugation and the protein concentration of the supernatant was determined, before use in the subsequent steps. MoDCs were resuspended in complete culture media with tumor cell lysates (1 mg of protein per 5 × 10^6^ MoDCs, per mL) and were incubated for 4 h at 37 °C, 5% CO_2_.

### Flow Cytometry Analysis

Cell surface staining was performed using monoclonal antibodies fluorescently labeled with fluorescein isothiocyanate (FITC), allophycocyanin (APC) or phycoerythrin (PE). Anti-CD3 and anti-CD14 antibodies (ImmunoTools GmbH, Friesoythe, Germany) were used to stain T cells and monocytes, respectively. Anti-CD86 antibody (ImmunoTools), and anti-HLA-DR (Immunostep; Salamanca, Spain) were used to assess MoDC maturation. Data were acquired with Attune® Acoustic Focusing Cytometer and analyzed using Attune® Cytometric Software v2.1 (Applied Biosystems, Carlsbad, CA, USA. In each case, at least 10,000 events were acquired in the gate of interest. The forward scattered (FSC) and the side scattered (SSC) light were used to evaluate relative cell size and granularity and cell doublets were excluded based on FSC-A/FSC-H. Mean Fluorescence Intensity (MFI) was determined for each marker.

### Co-Culture of MoDCs with Autologous T Cells

MoDCs were co-cultured with autologous T cells, using the respective CD14^−^ cell population (> 65% of T cells) that resulted from the monocyte isolation, which was thawed 1 day before co-culture. Cells were plated in a 96-well-round-bottom tissue culture plate in a 1 MoDC: 5 T cells ratio. After 1 week, the co-culture was re-stimulated with MoDCs and cultured for two more weeks. At the first day of co-culture and upon the MoDC re-stimulations, IL-7 (5 ng/mL) was added; IL-2 (10 IU/mL) was added always 1 day after IL-7. As a control, a condition with T cells alone was kept in parallel.

### Cytokine Detection

The cytokine production was quantified by enzyme-linked immunosorbent assay (ELISA) technique. Human interferon gamma (IFN-γ), TNF-α and IL-4 ELISA development kits (Immunotools) were used according to manufacturer’s instructions. Cytokine concentration was calculated using the specific standard curves.

### T Cell Cytolytic Capacity

T cell degranulation was evaluated by flow cytometric analysis of the cell surface expression of the lysosomal-associated membrane protein 1 (LAMP-1,CD107a), typically expressed in the membrane of cytotoxic granules [13–15].

Tumor cells were resuspended in complete DMEM media at a concentration of 1 × 10^5^ tumor cells/mL and then plated in a 96-well-flat bottom tissue culture plate and left in culture overnight, to allow the tumor cells to adhere to the bottom of the plate. The following day, all the supernatant was removed and replaced by the content of the wells of co-cultures of MoDCs (loaded or not with antigens) and T cells. Simultaneously, PE-conjugated anti-CD107a antibody (Abcam, Cambridge, UK) was added to each well. To facilitate de interaction between target and effector cells, the plate was centrifuged at 26×g for 30 s. After 1 h, Brefeldin A (BD Biosciences, NJ, USA) was added to each well and the plate was incubated at 37 °C for additional 4 h. Conditions with T cells and with tumor cells alone were cultured in parallel, as controls. After the incubation period, cells were harvested and analyzed in the flow cytometer. Using an FSC vs. SSC dot plot, the lymphocyte population was selected and the percentage of CD107a^+^ cells was calculated among them.

### Statistical Analysis

Statistical analysis was performed on experiments repeated in at least three independent assays (i.e cells collected from differentt donnors).. Experimental data were analyzed using GraphPad Prism version 6.01 (GraphPad Software, Inc.; La Jolla, CA, USA); statistical differences were determined using two-tailed paired Student’s *t* test or two-wayANOVA test for multiple comparisons. Data were presented as mean ± Standard Error of Mean (SEM). *p* values < 0.05 were considered statistically significant.

## Results

### Yield, Purity and Morphological Characteristics of Isolated Monocytes

To compare monocytes isolated by either MACS or EasySep cell separation technologies, PBMC samples were equally divided and processed simultaneously with one or the other technology. To determine the isolation yield, PBMCs and CD14^+^ cells were counted in the optic microscope, after each isolation step, and the number of CD14^+^ cells was divided by the initial number of PBMCs. The isolation yield was 6% significantly higher with MACS, which represent 25.00 ± 3.20% of the cells (*p* = 0.0371), while with EasySep we obtained 18.87 ± 2.69% (Table 1).

**Table 1:** Characteristics of monocytes isolated using the MACS or EasySep immunomagnetic isolation technologies

	MACS	EasySep
Isolation Yield (%)^a^	25.00 ± 3.20	18.87 ± 2.69 *
Purity (%) ^a^	94.98 ± 1.91	90.31 ± 2.50
Median FSC	3.49 × 10^6^ ± 0.04 × 10^6^	3.35 × 10^6^ ± 0.05 × 10^6^ **
Median SSC	4.04 × 10^6^ ± 0.15 × 10^6^	6.58 × 10^6^ ± 0.22 × 10^6^ ****

Values represent the Mean ± SEM values from 8 different donors. ^a^The isolation yield was calculated dividing the number of cells obtained after isolation by the number of cells prior to isolation (total PBMCs) × 100

FSC – forward scatter; SSC – side scatter

Statistical significance (*p < 0.05, **p < 0.01, ****p < 0.0001, ^a^ marginal significance 0.05 < *p* < 0.1) refers to differences between monocyte isolation using both methods

**Fig. 1:**
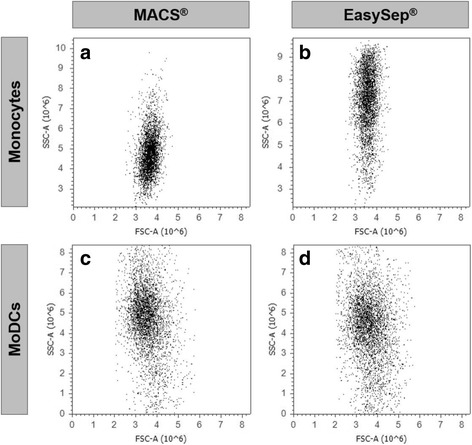
Comparison between monocytes and MoDCs obtained with MACS and EasySep technologies. **a** and **c** MACS technology (Miltenyi Biotec) and (**b**) and (**d**) EasySep (StemCell Technologies). FSC vs. SSC dot plot profiles, obtained by flow cytometry, are represented to assess cell size and complexity, respectively. A representative dot plot of one out of eight different donors is shown

To evaluate monocyte purity, cells were stained with anti-CD14 antibody and analyzed by flow cytometry. Monocyte purity was 90.31 ± 2.5% with EasySep and 94.98 ± 1.91% with MACS, a difference that was marginally significant (*p* = 0.0873, Table 1). Cell doublets, present in both samples, were very similar: 9.90 ± 2.32% and 11.96 ± 2.39% from MACS and EasySep, respectively and were excluded from the analysis. Moreover, it was also possible to observe differences in the FSC and SSC (Table 1 and Fig. 1). MACS monocytes showed an FSC of 3.49 ± 0.04 × 10^6^, which was significantly higher than the 3.35 ± 0.05 × 10^6^ observed with EasySep (*p* = 0.0044). On the other hand, EasySep monocytes showed an SSC of 6.58 ± 0.22 × 10^6^, which was significantly higher than the SSC of 4.04 ± 0.15 × 10^6^ observed in MACS monocytes (*p* < 0.0001). Hence, MACS monocytes showed a higher relative size, but decreased granularity, compared to EasySep monocytes. Concordantly, microscopically, we observed that EasySep presented more granules than the MACS monocytes (results not shown).

### MoDC Generation and Characterization

**Fig. 2:**
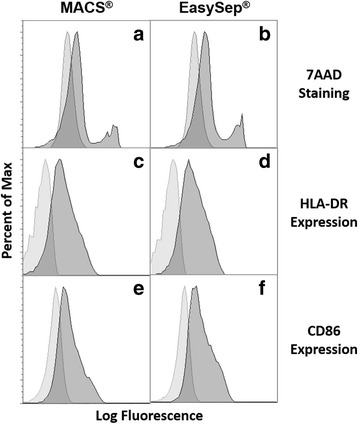
Comparison of MoDCs viability and maturation. After isolating monocytes with MACS or EasySep technologies, cells were cultured with GM-CSF and IL-4 during 1 week to allow their differentiation into MoDCs, as described in the Material and Methods section. MoDCs were collected and stained with different makers**:** 7AAD to assess cell viability (**a**, **b**); HLA-DR, a maturation marker (**c**, **d**); CD86, a co-stimulatory molecule (**e**, **f**). **a, c, e**– MoDCs derived from monocytes isolated with MACS technology; **b**, **d**, **f**– MoDCs derived from monocytes isolated with EasySep technology. Cells were analyzed by flow cytometry and the presented histograms refer to the population of stained MoDC (dark grey) and unstained control (light grey). A representative histogram of one out of eight different donors is shown

Monocytes obtained with both isolation kits were cultured with GM-CSF and IL-4 for 7 days to allow their differentiation into MoDCs. The resulting number of MoDCs were counted and the cell viability evaluated by 7AAD staining. The data showed that among MACS_MoDCs there were 14.04 ± 3.15% dead cells, while that percentage increased slightly to 22.17 ± 3.75% in EasySep_MoDC (*p* = 0.0731, Fig. 2a and b).

The differentiation efficiency, based on the percentage of monocytes that differentiated into MoDCs was calculated by dividing the number of viable collected MoDCs by the number of monocytes that had been initially plated. An average of 44.57 ± 3.63% of monocytes isolated with MACS differentiated into MoDCs, which was marginally higher than 35.85 ± 3.21% (*p* = 0.0776), the percentage of monocytes isolated with EasySep that differentiated into MoDCs (Table 2). To assess maturation, we compared the levels of expression of HLA-DR, which is the most expressed MHC-II molecule, and the costimulatory molecule CD86. The data showed that EasySep_MoDCs have higher expression of HLA-DR and CD86 (Fig. 2e and f, Table 2).

**Table 2:** MoDCs’ differentiation: efficiency, viability, maturation and co-stimulation markers

	MACS	EasySep
Differentiation efficiency (%)^a^	44.57 ± 3.63	35.85 ± 3.21 ^b^
7AAD (%)	14.04 ± 3.15	22.17 ± 3.75 ^b^
HLA-DR (MFI)	720.0 ± 100.0	995.8 ± 178.8

Values represent Mean ± SEM values from 5 different donors

^a^Differentiation efficiency was calculated based on the percentage of monocytes that differentiated into MoDCs, calculated by dividing the number of viable MoDCs collected by the number of monocytes that had been initially plated

^b^Marginal significance (0.05 < *p* < 0.1) refers to differences between monocyte isolation using both methods

### Response to Tumor Cell Antigens and Maturation Stimuli

**Fig. 3:**
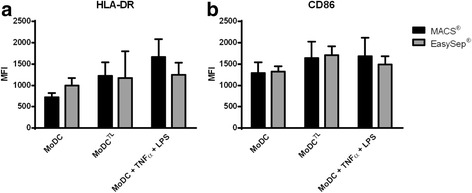
Comparison of activation level between MoDCs and responsiveness to maturation stimuli or tumor antigens. MACS_MoDCs (black bars, MACS) and EasySep_MoDCs (grey bars, EasySep) were loaded with tumor cell lysates (TL) from MCF-7 breast cancer cell line or stimulated with LPS and TNF-α during 4 h and analyzed by flow cytometry for the expression of (**a**) HLA-DR, and (**b**) CD86. Unstimulated MoDCs were used as controls. Data shown refer to mean ± SEM of MFI (*n* = 3 different donors)

Then we evaluated MoDCs’ capacity to respond to tumor antigens and to maturation stimuli. For this purpose, we challenged them with whole tumor cell antigens or with TNF-α plus LPS and compared the expression of HLA-DR and CD86. As shown in Fig. 3a, MACS_MoDCs when challenged with tumor antigens or maturation stimuli showed an increased expression of HLA-DR (1246.0 ± 286.3 or 1668.0 ± 412.8 MFI, respectively), compared to unstimulated MoDCs (720.0 ± 100.0 MFI). However, EasySep_MoDCs challenged with tumor cell antigen or maturation stimuli, showed a modest increase (1169.0 ± 632.5 or 1225.0 ± 316.3 MFI, respectively), compared to unstimulated MoDCs (995.8 ± 178.8 MFI).

MACS_MoDCs challenged with either whole tumor cell antigens or with TNF-α plus LPS showed also higher CD86 expression (1639.0 ± 389.8 and 1682.0 ± 435.5 MFI respectively), compared with unstimulated MoDCs (1291.0 ± 251.6 MFI) (Fig. 3b). However, in EasySep_MoDCs the stimulation led to a minor increase in CD86 expression (1707.0 ± 210.7 or 1491.0 ± 192.7 MFI, respectively), compared with unstimulated MoDCs (1319.5 ± 130.1 MFI).

The increase in maturation after either stimulus is less evident with EasySep than with MACS, when compared to unstimulated MoDCs. This suggests that EasySep_MoDCs are less responsive to maturation stimuli, compared to MACS_MoDCs.

### Capacity of MoDCs to Induce Cytokine Secretion by T Cells

**Fig. 4:**
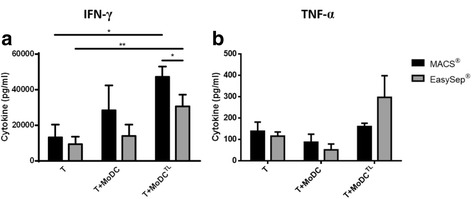
Comparison of cytokine secretion by T cells when co-cultured with tumor antigen-loaded MoDCs. T cells were cultured with autologous MoDCs (1 MoDC: 5 T cell) loaded or not with whole tumor cell lysates (TL) from MCF-7 breast cancer cell line. Control conditions with unstimulated T cells were run in parallel. After 3 to 5 days in co-culture, cytokine expression was evaluated by ELISA technique: (**a**) IFN-γ expression; (**b**) TNF-α expression. Black bars represent data obtained from T cells stimulated with MACS_MoDCs and grey bars data related to EasySep_MoDCs. Data refers to the mean cytokine secretion (pg/mL) ± SEM from 3 to 4 different donors. Asterisks represent statistical significance (*p < 0.05; **p < 0.01)

To compare the capacity to stimulate T cells, MACS_MoDCs and EasySep_MoDCs were loaded or not with whole tumor antigen and co-cultured with autologous T cells. After 3–5 days, supernatants were analyzed by ELISA for the presence of IFN-γ, TNF-α and IL-4. In general, we observed that T cells stimulated with either MACS or EasySep MoDCs loaded with tumor antigens express significantly higher levels of IFN-γ than unstimulated T cells (Fig. 4a). Yet, T cells stimulated by MACS_MoDCs secreted higher levels of IFN-γ. This difference was significant when considering the levels of IFN-γ expressed by T cells stimulated with tumor antigen-loaded MoDCs (*p* = 0.0485). In fact, MACS_MoDCs induced the secretion of approximately 47206 ± 5680 pg/mL of IFN-γ by T cells, while EasySep_MoDCs induced only 30598 ± 6559 pg/mL.

T cells stimulated with either MACS or EasySep_MoDCs loaded with tumor antigens showed a slight increase in TNF-α expression, compared with unstimulated T cells. This increase was higher with EasySep_MoDCs, compared to MACS_MoDCs (Fig. 4b). IL-4 secretion by T cells was not detected in any case (data not shown).

### Capacity of MoDCs to Induce T Cell Cytotoxicity towards Tumor Cells

To assess the capacity of MoDCs to induce antitumor activity, autologous T cells were co-cultured with the MoDCs loaded with MCF-7 tumor cells whole lysates. Then these T cells were used against the same viable tumor cell line. Upon stimulation, cytotoxic T cells release lytic granules; bringing their content to the cell membrane. Therefore, we analyzed the presence at the cell surface of the molecule CD107a expressed in lytic granules, to determine the percentage of T cells activated against tumor cells.

**Fig. 5:**
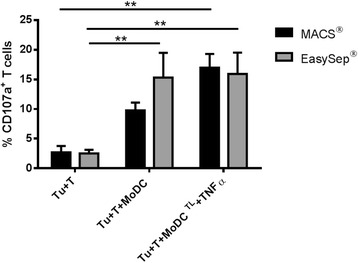
T cell degranulation upon challenge with tumor cells. MoDCs from HLA-A*02 donors were loaded with MCF-7 whole tumor cell lysates (TL). Then, these MoDCs were used to stimulate autologous T cells. Co-culture took place for 3 weeks (1 MoDC: 5 T cells), with a MoDCs re-stimulus after 1 week of co-culture. Each set of MoDCs (MACS or EasySep) was cultured with the respective CD14^−^ fraction. After 3 weeks, T cells were co-cultured with the MCF-7 breast tumor cell line for 5 h, and T cell degranulation was assessed by flow cytometry. Control conditions with tumor cells and unstimulated T cells (Tu + T) and tumor cells with T cells stimulated with unloaded MoDCs (Tu + T + MoDC) are also presented in the graph. Data refers to the mean percentage of CD107a^+^ cells ± SEM from 4 different donors. Asterisks represent statistical significance (**p < 0.01)

As shown in Fig. 5, the percentage of degranulating (CD107a^+^) T cells showed a small increase from 2.7 ± 1.1% for unstimulated T cells to 9.8 ± 1.3% when co-cultured with MACS_MoDCs but increased significantly from 2.5 ± 0.6% to 15.3 ± 4.2%, with EasySep_MoDCs. When MoDCs were loaded with tumor antigens, the percentage of degranulating T cells increased to 15.5 ± 2.5% with MACS and to 17.0 ± 1.7% with EasySep. MoDCs So, comparing the antigen-specific degranulation, i.e., the percentage of degranulating T cells after challenge with antigen-loaded MoDCs minus the respective values obtained with unloaded MoDCs, MACS_MoDCs induce an average of 5.8% specific degranulating T cells, while EasySep_MoDCs induce only 1.7% of antigen-specific degranulating T cells.

## Discussion

The most common process to obtain human DCs for vaccination purposes is to differentiate them from monocytes, i.e., MoDCs [16]. The production of MoDCs requires challenging and laborious task with many different cell manipulation steps, from blood sample collection to monocyte isolation and DC culture. These cell manipulation steps may affect cell viability, recovery, and function and therefore require optimization [17–20]. Thus, understanding which monocyte isolation methods allows the highest cell yield and purity and MoDCs with the capacity to respond to antigen stimulation and activate T cells is important [16, 18, 19, 21]. To address these questions, we compared the two most used positive selection immunomagnetic monocyte isolation methods: the MACS, and EasySep technologies.

As shown in the results, MACS technology allowed a significantly higher isolation yield, as well as a higher purity, compared to EasySep. Interestingly, in another study where these two technologies were compared for the isolation of osteoprogenitor cells, using the mesenchymal stem cell antigen-1, the MACs technology also resulted in a higher purity [22]. Yet, this is the first report demonstrating differences in yield and purity of monocytes obtained by either method.

Monocytes isolated with EasySep technology are significantly more complex, as shown by the higher side scattered (SSC) light upon flow cytometry analysis. This difference may be a consequence of internalization of EasySep beads [23]. In fact, when we decrease the amount of beads used in the isolation (maintaining a constant cell number), the SSC of the isolated cells also decreased (data not shown). Both observations support the idea that the higher monocyte complexity is probably caused by the internalization of the EasySep beads. It is worth noting that the incubation of mononuclear cells with MACS beads takes place at 4 °C while with EasySep, it is performed at room temperature, which favors endocytosis by monocytes.

Monocytes isolated with EasySep retrieved less dendritic cells, which may be explained by the higher percentage of cell death during differentiation into MoDCs. Since monocytes isolated with EasySep present an initial more complex profile, this may mean that they are biologically more altered, which may contribute to the activation of some of the several mechanisms involved in monocyte death [24, 25]. Given the relevance of immune cell apoptosis in homeostasis and disease [26, 27], further studies are recommended to access the inherent mechanisms behind EasySep monocytes death.

By comparing the expression of maturation markers in both sets of MoDCs we observed that EasySep_MoDCs have a more mature profile. Mucci et al. [23] suggested that this initially more activated state of EasySep_MoDCs is related to the composition of EasySep beads; whose dextran-coating would be more easily captured by mannose receptors and therefore trigger intracellular signaling. However, the beads from MACS are also composed of dextran aggregates, and can potentially be recognized by mannose receptors [28]. Hence, we suggest this higher EasySep_MoDC maturation may be due to a internalization mechanism independent of mannose receptors. Nonetheless other aspects could also have contributed to the observed differences such as, the antibody used in MACs and EasySep and the influence of the column in MACS separation or TAC particles in EasySep separation. Further studies would be advised to better define the influence that both technologies have on cells.

To assess the influence of MoDCs in T cells’ function, both sets of MoDCs, previously loaded with whole tumor cell lysates were used in co-culture with autologous T cells. Our results showed a higher secretion of IFN-γ and TNF-α by T cells stimulated with antigen-loaded MoDCs, consistent with a Th1 response resultant from both MACS and EasySep processed cells. Nevertheless, IFN-γ secretion was significantly higher with MACS. On the other hand, TNF-α secretion was higher in the EasySep co-culture, although there was no significant difference between the two technologies. IL-4 expression was below detection levels in both cases. It has been previously described by Elkord et al. [7] that cytokine production by MoDCs is influenced by the monocyte isolation method; they suggested that the microbeads by linking to the CD14 receptor at monocyte surface could block it, preventing the secretion of some cytokines upon the stimulus.

Average isolation time with MACS technology was slightly longer due to the elution steps; whose duration depends on the velocity of cell passage through the magnetic column [29]. Since autologous T cells, used for the co-culture assays, were obtained from the total negative fraction obtained upon monocyte isolation, one could argue that the isolation method had an influence on T cells too. However, the data obtained for unstimulated T cells showed no differences in T cell function and make us confident that the differences are solely due to the presence MoDCs.

When assessing the T cell cytotoxicity against tumor cells we observed an improved antigen-specific degranulation of T cells that have been stimulated with MACS_MoDCs loaded with tumor cell lysates. In the case of T cells stimulated by EasySep_MoDCs, a significant T cell degranulation is already observed in the presence of dendritic cells alone, which indicates a response that is not antigen-specific. The differences observed in the antigen-specific T cell activation may be attributed to the fact that unloaded EasySep_MoDCs show already a significant level of maturation which may contribute to exhaustion and lack of responsivity to stimulation.

## Conclusions

To be used as therapeutic anti-cancer vaccines, MoDCs must be obtained in sufficient numbers and with high capacity to respond to tumor antigen stimulation and undergo proper maturation. If these requisites are met, mature MoDCs will be able to effectively activate effector T cells meeting the purpose of DC-based anti-cancer immunotherapies. Here we have assessed the influence of the two most used monocyte isolation methods, the immunomagnetic MACS and EasySep technologies in the functionality of dendritic cells derived from the monocytes obtained from both techniques.

We have concluded that both kits allow the isolation of monocytes with high purity, though MACS technology results in higher cell yields. Moreover, T cells stimulated by MoDCs processed with MACS express significantly higher levels of IFN-γ and display higher antigen-specific degranulation, indicating this is more suited to be used in the production of MoDCs with antitumor immunogenicity.

## Abbreviations

7AAD: 7-amino-actinomycin D
DCs: Dendritic cells
FSC: Forward scatter
HLA-DR: Human Leukocyte Antigen – antigen D Related
LPS: Lipopolysaccharide
MFI: Mean fluorescence intensity
MoDCs: Monocyte-derived dendritic cells
PBMCs: Peripheral blood mononuclear cells
SSC: Side scatter
TL: Tumor lysate
